# Work location choice- the perspective of graduates: Survey dataset in Vietnam

**DOI:** 10.1016/j.dib.2021.106788

**Published:** 2021-01-22

**Authors:** Thuy Thu Nguyen, Thi Phuong Linh Nguyen, Thi Thanh Hoa Phan, Trong Nghia Vu

**Affiliations:** National Economics University, Vietnam

**Keywords:** Workplace Decision, Place Attractiveness, Family Support, Social Norm, Graduates, Return Hometown, Human Resource Management

## Abstract

How to attract knowledge human resources, namely graduate students for the economics development of the rural regions is a big question for any government. The patterns of young graduates’ move during the transition from universities to the labour market: stay in the big city or return back to their rural hometown to begin their work life, why do people return rural hometown to settle their careers, are needed to be deeply considered in various contexts. The data represents a quantitative study to assess the relationship between working location choice of graduate and its determinants in Vietnam – a developing country context. This data consists of the demographic characteristics of the sample and two main variables. First, place attractiveness with three sub-dimension including quality of life, job opportunities, and place attachment. Second, social dimension with two sub-dimension which are social norms and family supports. The data were collected using a cross-sectional questionnaire and was analysed using SPSS version 22. Analyses of the data can provide insights into determinants of return hometown to work decision that may be useful for researchers in the field to understand workplace choice of graduates, for business managers who want to attract high skilled staff and for rural communities and provincial policymakers. It may serve as a reference to expand research and to develop interventions to encouraging student return back hometown to work after university graduation.

**Specifications Table**

**Table:** 

Subject	Human Resource Management
Specific subject area	Workplace choice after graduated from higher education
Type of data	Table Excel file
How data were acquired	Data were collected through a survey as a self-administered questionnaire developed within a research project
Data format	Raw, analyzed, descriptive and inferential statistical data
Parameters for data collection	The target population of the questionnaire is graduate students who temporarily migrated to Hanoi - the capital of Vietnam for getting higher education and just graduated within 1–3 years from universities. Those people have made decision on return hometown or stay migrate in Hanoi for working after graduated
Description of data collection	The research questionnaire was designed by adaptation measures from previous researches for Vietnamese context. We conducted the data collection by using Google docs. We upload soft electronic copies of survey questionnaire online. The questionnaires were sent to about 1902 email addresses, which were collected from student alumni of 5 universities in Hanoi –the capital of Vietnam. We received 510 responses (response rate of 26.8%). After screening the questionnaires, bias answers were eliminated. The final sample size consists of 502 responses.
Data source location	5 universities in the North of Vietnam National Economics University, Hanoi University of Science and Technology, Vietnam National University, Foreign Trade University and Banking University
Data accessibility	Data and the questionnaire used in the survey are available in this article. and in Mendeley Data http://dx.doi.org/10.17632/cdx7kbw9t5.1

**Value of the Data**

• This data set can be beneficial to analyze the factors that determine the work location choice, the decision to return hometown to work or stay migration in city after graduated from higher education.
• The dataset will be useful for researchers who want to examine the impact of place attractiveness, family support and social norm on the college students’ working place decision. The data can be useful for business managers and public policy makers to attract knowledge workers.
• The data can be used by future research to further analyze other potential factors influencing work location choice among employees, develop a cross-country comparison model or apply other research methods in analyzing work location choice. The data on the work location choice of business and economics graduate can be compared to other samples in a different field of study including technical, engineering, health and education.
• This dataset may help inform reliability and validity values on the adapted instruments.

## 1 Data Description

Choosing the right career path is becoming more and more important for young students today [1]. Students have to take into account many things when choosing a career and working place location [2]. Many factors and people can influence a student's decision including social and cultural norm, parents, role models, friends in a student's life and attractiveness of locations [1], [2], [3]. Most of the time, the combination of social factors, job opportunities and quality of living environment are key determinants of working location choice [4], [2], [5].

The data can provide insight into the relations between social and perceived place attachment of individuals and working location choice. The survey data file spreadsheet accompanying this article consists of 503 rows and 28 columns. Each row represents an individual's response to a questionnaire. A five-point Likert range scale was used to allow the respondents to indicate how much they agree or disagree with a particular statement, so a numerical value in the data file means the respondent level of agreement, with 5 being "strongly agree" and 1 being "strongly disagree". Each questionnaire item in the columns was given a label, as shown in the first row. HL is the short form for quality of living environment; SN for social norm; JO for job opportunities; PA for place attachment; FS for family support. The measurement social norm includes three items of SN1, SN2 and SN3; four items of family support: FS1, FS2, FS3, FS4; five items of place attachment: PA1, PA2, PA3, PA4 and PA5; three items of Job opportunities: JO1, JO2, JO3 and seven items for Quality of living environment: HL1, HL2, HL3, HL4, HL5, HL6 and HL7. (See Table 1 below). Two purposes are expected to achieve by utilizing the survey data. The first is to examine the settlement intention of the interviewees and corresponding reasons. The second is to analyse the place attractiveness and social influencing factors on their settlement decision-makings.

**Table 1:** Label of data.

Constructs	Items	Code
Job opportunities	How easy would it be for you to find a job in your hometown that is as good as the one in Hanoi?	JO1
	How easy would it be for you to find a job in your hometown that is much better than the one in Hanoi?	JO2
	How easy would it be for you to find a job in your hometown that is much better than the one in other places?	JO3
Place attachment	I would feel relaxed if I am at my hometown.	PA1
	I would feel happiest if I am at my hometown.	PA2
	My preferred living environment would be my hometown	PA3
	I usually visit my hometown if I have free time	PA4
	I would really miss my hometown if I am away from it for too long.	PA 5
Quality of living environment	Satisfaction with the quality and availability of housing in your hometown	HL1
	Satisfaction with the quality and availability of health care services in your hometown	HL2
	Satisfaction with the quality and availability of education and interest education in your hometown	HL3
	Satisfaction with the quality and availability of leisure time activities in your hometown	HL4
	Satisfaction with the quality and availability of culture in your hometown	HL5
	Satisfaction with the transportation in your hometown	HL6
	Satisfaction with the living conditions in your hometown	HL7
Subjective norms	Your parents encourage you to return hometown for working	SN1
	Your friends encourage you to return hometown for working	SN2
	The people who are important to you encourage you to return hometown for working	SN3
Family support measure	When I have a tough day, family members try to cheer me	FS 1
	Family members share family-related ideas and advice with me	FS 2
	If I am having problems, my family provides advice	FS 3
	I can depend on my family to help if I really need it	FS 4

(Source: authors’ survey)

Table 2 displays demographic statistics for the 502 respondents. Total 502 observations in the data set. In which, 57.2% are men. All the respondents live in the north of Vietnam. The respondent's place of living before migrating to Hanoi for higher education: 76.6% in rural areas, 23.4% used to live in other cities. 95% of the respondents’ parents still live in their hometown. 21.5% return to hometown after graduated from universities, 69.3% of the respondents stay in Hanoi for working after graduated, and 9.2% to other cities for working. Regarding age, 100% of the respondents are under 24 years old. A majority of respondents are undergraduate 90%, after 1 to 3 years graduated from university; 10% have highest level of education completed in masters’ degree (see Table 2).

**Table 2:** Demographic statistics.

TT	Demographic statistics	Frequency (persons)	Percentage (%)
1	Place of birth	502	100%
	Urban	125	24.9
	Rural	377	75.1
2	Gender		
	Male	287	57.2
	Female	215	42.8
3	Parents’ in hometown		
	Stay in hometown	476	94.8
	Migrate to another place	26	5.2
4	Academic performance		
	Fair	316	62.9
	Good	124	24.7
	Excellent	62	12.4
5	Working Experience during university		
	No	106	21.1
	Yes	393	78.3
6	Working place choice		
	Hanoi	348	69.3
	Other urban cities	46	9.2
	Hometown	108	21.5

(Source: authors’ survey)

Table 3 show that only 21.5% of respondent return hometown to work after graduated from universities. Most of them stay in Hanoi for job. 9.3% of respondents find job opportunities in another urban city. 43.5% graduates who return hometown to work after graduation are female. Only 11.5% of graduates whose family have migrated from their hometown decided to come back to their hometown for working, most of them (89.5%) return if their families still live in there.

**Table 3:** Return decision: Parents and gender differences.

		Count				
		Parents		Gender		
		Live in hometown	Move away	Female	Male	Total
Return	.00	371	23	168	226	394
	1.00	105	3	47	61	108
Total	476	26	215	287	502	

(Source: authors’ survey)

19% students whose place of origin in rural areas decided to return hometown for working while the number in urban respondents is a little higher of 28.8%. Students with working experience during school time also tend to return hometown (36%) while only 17% of students without working experience during school time would return hometown to work (Table 4).

**Table 4:** Cross tabulation.

		Count				
				Part time work		
		Place of birth		during university		
		Urban	Rural	Yes	No	Total
Return	.00	89	305	68	323	394
	1.00	36	72	38	70	108
Total	125	377	106	393	502	

(Source: authors’ survey)

Table 5 provides information on the variables through descriptive statistics mean, standard deviation and variance.

**Table 5:** Descriptive statistics items.

	Descriptive statistics			
	N	Mean	Std. deviation	Variance
SN1	502	3.259	1.2140	1.474
JO1	502	2.956	1.0657	1.136
JO2	502	2.733	1.1036	1.218
JO3	502	2.544	1.1413	1.302
YQ1	502	4.060	.9563	.915
YQ2	502	3.805	1.0768	1.159
YQ3	502	3.637	1.0980	1.206
SN2	502	2.709	1.0662	1.137
SN3	502	3.315	1.1990	1.438
YQ4	502	3.892	1.0287	1.058
YQ5	502	4.090	.9675	.936
HL1	502	3.759	.9476	.898
HL2	502	3.078	.9990	.998
HL3	502	3.143	.9641	.929
HL4	502	3.600	1.0444	1.091
HL5	502	3.554	.9707	.942
HL6	502	3.382	.9609	.923
HL7	502	2.990	1.0099	1.020
FS1	502	3.376	1.2932	1.672
FS2	502	2.831	1.1741	1.378
FS3	502	2.775	1.1509	1.324
FS4	502	2.531	1.1142	1.242

(Source: authors’ survey)

Table 6 provides information on the validity of the variables and factor loadings (factor correlation coefficients). EFA analysis at the same time for 22 items of 5 independent variables with varimax rotation method loaded in 6 factors. Almost items loaded in its original factor with factor loading in all cases above 0.5 except HL3 and HL1 loaded in wrong group. After Cronbach's Alpha analysis and consider the variable content with qualitative study, HL1 item of the "quality of living" measure have been eliminated. After the reduction of HL 1 item, EFA was conducted again with 21 items, the result support the validity of the measurement instruments. All items are then loading in five groups, on the factors appropriate to the original variables as shown the Table 6. The total variance explained by these five factors is 69.041%; KMO – Meyer = 0.841 (>0.6); Eigenvalue >1 [6]. Overall, KMO and Bartlett's Test value also suggest the suitability of structure detection. Table 6 shows an overview of the rotated component matrix, all items below 0.3 were cut off to better visualize which components the variables are loading on. The factor loadings indicate a high correlation, the correlation between the factor and the variables are highest 0.859 and lowest 0.573. All the item loadings are high (> 0.5) on its parent factor with low cross loading (< .3) on the other, then we can assume the convergent and discriminant validity of all scales [6]. After all, the analysis provides evidence to support the validity of the measurement instruments.

**Table 6:** Rotated component matrix and Cronbach's Alpha of measurements.

	Component					
	1	2	3	4	5	Cronbach's Alpha
HL6	.841					0.861
HL5	.811					
HL7	.718					
HL3	.687					
HL4	.656					
HL2	.573					
YQ2		.822				0.835
YQ1		.808				
YQ3		.763				
YQ5		.690				
YQ4		.575				
FS2			.859			0.871
FS4			.843			
FS3			.804			
FS1			.772			
JO2				.847		0.854
JO1				.813		
JO3				.765		
SN1					.842	0.804
SN3					.822	
SN2					.675	

Extraction Method: Principal Component Analysis.

Rotation Method: Varimax with Kaiser Normalization.

a. Rotation converged in 6 iterations.

(Source: authors’ survey)

Cronbach's Alpha analysis for independent and dependent variables proved that all variables are reliable with the minimum Cronbach's Alpha of 0.804. The Cronbach's alpha of the constructs relating to perceived quality of living environment is 0.861. Cronbach's Alpha of subjective norms is 0.804, place attachment 0.835 and job opportunities is 0.854; family support 0.871; thus, all exceeding the 0.70 cut-off value for reliability consistency (Hair et. al., 2006) [6]; all the value of “Corrected item total correlation" are more than 0.3 and all the research variables have “Cronbach's Alpha if item deleted” are lower than its overall Cronbach's Alpha. Therefore, all variables are proved to be internally consistent scales and reliable.

Table 7 show the high level of perceived place attachment in this sample group (mean = 3.897), expressing the hometown love of Vietnamese people. Graduate perceived opportunities for a good job is quite low in their rural hometown compared to big city like Hanoi (mean = 2.71). Supports from family for settling in hometown is also low (mean = 2.87).

**Table 7:** Statistics of scales.

Statistics	Social norm	Place attachment	Family support	Job opportunities	Quality of living environment
Mean	3.0943	3.8976	2.8790	2.7191	3.3538
Std. Deviation	.98490	.79023	1.00618	.90568	.77347
Variance	.970	.624	1.012	.820	.598

(Source: authors’ survey)

The data provided in this article contains two supplemental files:

The pdf-File “Questionnaire” shows a printout of the survey design as it has been presented to the survey participants.

The MS Excel-file “Data Set” contains the raw data of questionnaire responses from the conducted survey.

## 2 Experimental Design, Materials and Methods

### 2.1 Questionnaire development

We first reviewed carefully literature to find scales from previous research and then translated them into Vietnamese. The survey questionnaire was designed by borrowing measures from previous research. Those measure have been adapted for use in Vietnam context. The measure quality of living environment includes of 7 items adapted from Ezmale (2012) research [7]. This scale use five-point Likert scale, from 5 (totally satisfied) to 1 (totally no satisfied). Jorgensen & Stedman (2001) [8] measurement of place attachment with 5 items were borrowed; family support includes 4 items from Boyar et al. (2014) [9]; Soon (2010) [4] scale of job opportunities was used with 3 items; and social norms includes 3 items adapted from Linan and Chen (2006) and Boyar et al. (2014) research [9,10]. Those measures use five-point Likert scale, from 5 (strongly agree) to 1 (strongly disagree). Questionnaires were first translated into English. Then, two English experts checked the translations and translate them back to English to compare the two version. Minor revisions were made due to some differences in wording between the original version and the back translated version. We pretested the questionnaire by interviews in qualitative study. 5 in - depth interviews were conducted with 5 graduate students in March 2018 to revise, double check the measurements, in order to ensure that the questionnaire is usable with Vietnamese context before using this survey instrument for the official quantitative study. Each interview lasted about 1 hour. Then, we made necessary changes to ensure the questionnaire are accurate and understandable before final revision and administration in the study.

### 2.2 Questionnaire content

The questionnaire included 22 items and it took about 5 min to complete. The questionnaire consists of 2 parts.

The first part consists of questions asking about perceived personal point of view of each quote. The respondents were asked to state their agreement and disagreement on a 5-point Likert scale (5 - strongly agree to 1 - strongly disagree).

The second part consist of questions on demographic profile of the respondents. Demographic information and contextual factors were collected to obtain additional information regarding the targeted sample. Students’ academic performance, gender, place of origin, parent locations were included in the research. In addition to those demographic variables, respondents were asked about their current working place after graduated from universities.

### 2.3 Data collection

The subjects for this study are graduated students with place of origin in rural or other provincial cities other than Hanoi- the capital of Vietnam. Upon graduating from higher education institution and entering first employment, a graduate must make a decision on whether he/she would stay in Hanoi or move to another place or return hometown for a job. We focused on a sample of graduates who newly graduated 1–3 years from the schools. We conducted the data collection via Google docs. The soft electronic copies of survey questionnaire were uploaded online to alumni, class group, association Facebook accounts, (I got addresses' information from my old students and faculty alumni). I asked and reminded class speakers or class headers to ask graduated students in their class to answer the questionnaire. The questionnaires were sent to about 1902 email addresses getting from alumni of 5 big universities which located in Hanoi capital, the north of Vietnam. 510 answer sheets were received (response rate of 26.8%). After collecting the questionnaires, the data was checked to ensure that the sample consist of the research designed subjects. 5 responses were eliminated because they had been answered by students, who were born in Hanoi - inappropriate survey subjects. I eliminated additional 3 questionnaires due to their inconsistent or bias answers. After screening the questionnaires, the final sample size is of 502 responses.

Data analysis procedures comprise statistical analyses of the questionnaire data with the computer software Statistical Package for the Social Sciences (SPSS) version 22.

## Ethics Statement

This study was a voluntary survey. The responses were fully anonymous. The authors kept to all ethical concerns during the data gathering process and ensured that all information was used for research purposes and was absolutely confidential. We confirm that informed consent of all participants has been obtained.

## CRediT Author Statement

**Thuy Thu Nguyen:** Conceptualization, Methodology Writing Original draft preparation, Writing Reviewing & Editing, Supervision; **Thi Phuong Linh Nguyen:** Software Investigation; **Thi Thanh Hoa Phan:** Visualization, Investigation; **Trong Nghia Vu:** Funding acquisition, Validation, Resources.

## Declaration of Competing Interest

The authors declare that they have no known competing financial interests or personal relationships which have, or could be perceived to have, influenced the work reported in this article.

## Appendix Supplementary materials

Image, application 1

Image, application 2
