# Supplementary material for: Work location choice- the perspective of graduates: Survey dataset in Vietnam
Source: Data Brief. 2021 Jan 22;35:106788. doi: 10.1016/j.dib.2021.106788 (PMC7851764; doi:10.1016/j.dib.2021.106788)
Supplement: Supplementary file 1 [file mmc1.doc]

**Thuy T. Nguyen :** Conceptualization, Methodology, Writing- Original draft preparation, Writing- Reviewing and Editing, Supervision. **Linh P.T. Nguyen**: Software, Investigation. **Hoa T.T. Phan**: Visualization, Investigation. **Nghia T. Vu**: Funding acquisition, Validation, Resources.
