# Supplementary material for: Work location choice- the perspective of graduates: Survey dataset in Vietnam
Source: Data Brief. 2021 Jan 22;35:106788. doi: 10.1016/j.dib.2021.106788 (PMC7851764; doi:10.1016/j.dib.2021.106788)
Supplement: Supplementary file 2 [file mmc2.pdf]

QUESTIONAIRE

PART 1

1. Indicate your level of agreement with the following sentences from 1 (total disagreement) to 5 (total agreement). -

No	Items	Totally disagree	Disagree	Neutral	Agree	Agree
1	Your parents encourage you to return hometown for working	☐1	☐2	☐3	☐4	☐5`
2	How easy would it be for you to find a job in your hometown that is as good as the one in Hanoi?	☐1	☐2	☐3	☐4	☐5`
3	How easy would it be for you to find a job in your hometown that is much better than the one in Hanoi?	☐1	☐2	☐3	☐4	☐5`
4	How easy would it be for you to find a job in your hometown that is much better than the one in other place?	☐1	☐2	☐3	☐4	☐5`
5	I would feel relaxed if I am at my hometown.	☐1	☐2	☐3	☐4	☐5`
6	I would feel happiest if I am at my hometown.	☐1	☐2	☐3	☐4	☐5`
7	My preferred living environment would be my hometown	☐1	☐2	☐3	☐4	☐5`
8	Your friends encourage you to return hometown for working	☐1	☐2	☐3	☐4	☐5`
9	The people who are important to you encourage you to return hometown for working	☐1	☐2	☐3	☐4	☐5`
10	I usually visit my hometown if I have free time	☐1	☐2	☐3	☐4	☐5`
11	I would really miss my hometown if I am away from it for too long.	☐1	☐2	☐3	☐4	☐5`
12	When I have a tough day, family members try to cheer me	☐1	☐2	☐3	☐4	☐5`
13	Family members share family-related ideas and advice with me	☐1	☐2	☐3	☐4	☐5`

14	If I am having problems, my family provides advice	☐1	☐2	☐3	☐4	☐5`
15	I can depend on my family to help if I really need it	☐1	☐2	☐3	☐4	☐5`

2. *Evaluate your satifaction with the following living conditions of your hometown*

TT		EXTREMELY NO SATISFIED	SLIGHTLY NO SATISFIED	NEUTRAL	SATISFIED	EXTREMELY SATISFIED
16	Satisfaction with the quality and availability of housing in your hometown	☐1	☐2	☐3	☐4	☐5`
17	Satisfaction with the quality and availability of health care services in your hometown	☐1	☐2	☐3	☐4	☐5`
18	Satisfaction with the quality and availability of education and interest education in your hometown	☐1	☐2	☐3	☐4	☐5`
19	Satisfaction with the quality and availability of leisure time activities in your hometown	☐1	☐2	☐3	☐4	☐5`
20	Satisfaction with the quality and availability of culture in your hometown	☐1	☐2	☐3	☐4	☐5`
21	Satisfaction with the transportation in your hometown	☐1	☐2	☐3	☐4	☐5`
22	Satisfaction with the living conditions in your hometown	☐1	☐2	☐3	☐4	☐5`

PART 2: GENERAL INFORMATION

1. *Gender:* ◻ Male ◻ Female
2. *Your academic performance*

   ◻ Fair ◻ Good ◻ Excellent

3. *Place of living*

□ Hanoi □ Other provinces

4. Are your parents still linving in your hometown

□ Yes □ No

5 Where are working now

□ Hanoi □ Hometown □ Other place

6. Did you have part time job during your school time at your university

□ Yes □ No

Thank you
